# Establishment and molecular characterization of novel luminal A and luminal B canine mammary cancer cell lines for comparative oncology

**DOI:** 10.14202/vetworld.2025.1725-1740

**Published:** 2025-06-26

**Authors:** Juthathip Jurutha, Yanika Piyasanti, Kornkanok Sritabtim, Suparat Chaipipat, Kannika Siripattarapravat, Sukumal Prukudom, Usuma Jermnak, Rungthiwa Sinsiri, Kakanang Wongsuppabut, Charuwan Wongsali, Nutawan Niyatiwatchanchai, Wijit Sutthiprapa, Napachanok Swainson, Wachiraphan Supsavhad

**Affiliations:** 1 Center for Veterinary Diagnostic Laboratory, Bangkhen, Faculty of Veterinary Medicine, Kasetsart University, Bangkok, 10900, Thailand; 2 Department of Pathology, Faculty of Veterinary Medicine, Kasetsart University, Bangkok, 10900, Thailand; 3 Department of Anatomy, Faculty of Veterinary Medicine, Kasetsart University, Bangkok, 10900, Thailand; 4 Department of Pharmacology, Faculty of Veterinary Medicine, Kasetsart University, Bangkok, 10900, Thailand; 5 Operation and Laparoscopic Center, Kasetsart University Veterinary Teaching Hospital, Faculty of Veterinary Medicine, Kasetsart University, Bangkok, 10900, Thailand; 6 Department of Biochemistry, Faculty of Science, Kasetsart University, Bangkok, 10900, Thailand

**Keywords:** canine mammary cancer, cell line establishment, comparative oncology, immunohistochemistry, luminal A, luminal B, metastasis, molecular subtype, reverse transcription quantitative real-time polymerase chain reaction

## Abstract

**Background and Aim:**

Canine mammary cancer (CMC) is the most frequently diagnosed malignancy in female dogs, sharing significant pathological and molecular similarities with human breast cancer (HBC). Despite the availability of various CMC cell lines, most represent triple-negative orepidermal growth factor receptor 2 (ErbB2)-enriched subtypes, which limit research on hormone receptor-positive cancers. This study aimed to establish and characterize novel CMC cell lines representing luminal A and B subtypes.

**Materials and Methods:**

Between 2020 and 2021, 31 canine mammary tumors (CMTs) were collected from clinical cases. Tumor tissues were processed for primary culture, and two cell lines – CMGT_071020 and CMGT_180321 – were successfully established. Immunohistochemistry (IHC) was used to assess expression of estrogen receptor alpha (ERα), progesterone receptor (PR), ErbB2, Ki-67, vimentin, and multi-cytokeratin. Functional assays (wound-healing and transwell migration) assessed metastatic behavior. Gene expression (*EGFR*, *TP53*, *Bcl-2*, *PTEN*, *SNAIL*, *N-cadherin*, and *E-cadherin*) was analyzed using reverse transcription quantitative real-time polymerase chain reaction (RT-qPCR). Cell line authentication was confirmed through short tandem repeat (STR) profiling and mycoplasma testing.

**Results:**

The CMGT_071020 (luminal B) and CMGT_180321 (luminal A) cell lines were derived from malignant epithelial tumors and maintained stable growth over 30 passages. IHC confirmed molecular subtype classifications. CMGT_071020 exhibited a fibroblast-like morphology, a high Ki-67 index (67%), and superior migratory capacity compared to CMGT_180321 and the commercial ErbB2-enriched REM134 cell line. *E-cadherin* expression was significantly elevated in CMGT_071020 (*p* < 0.05), whereas the expression levels of other genes were comparable. STR analysis verified their genetic uniqueness, and both lines were free from mycoplasma contamination.

**Conclusion:**

This study successfully established and characterized two novel hormone receptor-positive CMC cell lines, representing luminal A and luminal B subtypes. The CMGT_071020 line exhibited higher metastatic potential, offering a promising model for aggressive hormone-responsive CMC. These cell lines provide valuable tools for comparative oncology and may facilitate subtype-specific therapeutic research.

## INTRODUCTION

Canine mammary cancer (CMC) represents the most frequently diagnosed neoplasm in non-spayed female dogs and shares several clinical and pathological parallels with human breast cancer (HBC). According to the Veterinary Society of Surgical Oncology [1], CMC accounts for over 40% of all canine malignancies. The mortality rate associated with CMC has been rising steadily, with data indicating that CMC and skin cancers are among the two leading causes of death in dogs [2]. The development of canine mammary tumors (CMT), encompassing both benign and malignant forms, is influenced by various factors, including age, breed, genetic predisposition, and hormonal status. Notably, more than 60% of CMT cases are reported in senior purebred dogs [3].

Genetic mutations play a substantial role in tumor development. Several genes have been implicated in CMT progression, including cyclooxygenase-*2* (*COX-2*), vascular endothelial growth factor (*VEGF*), myelocytomatosis (*MYC*), tumor suppressor p53 (*TP5*3), phosphatase and tensin homolo*g* (*PTEN*), and B-cell lymphoma *2* (*Bcl-2*) [4–12]. In particular, dysregulation of tyrosine kinase receptors, such as epidermal growth factor receptor 1 (*ERBB1/EGFR*) and epidermal growth factor receptor 2 (*ERBB2/ErbB2*) activates key signaling pathways involved in tumor cell proliferation and metastasis [13, 14]. Hormonal influences also play a significant role in CMT pathogenesis; prolonged exposure to estrogen and progesterone can disturb cellular homeostasis and initiate oncogenic processes [15, 16].

At present, mastectomy and ovariohysterectomy remain the most reliable therapeutic options. However, recurrence rates remain high, rising from 58% following the initial surgery to 77% after secondary interventions. Furthermore, approximately 75% of recurrent tumors exhibit increased malignancy compared to their primary counterparts [17–19]. Although chemotherapy is available for systemic treatment, it is often associated with adverse effects such as pyometra, vaginal bleeding, and anemia [20].

Given these limitations, the development of more effective and subtype-specific therapies is imperative. Precision medicine tailored to the molecular profile of individual CMC subtypes may offer improved therapeutic efficacy. CMC and HBC share numerous similarities in their pathological and molecular features [21–23]. HBC is classified into four major subtypes based on immunohistochemical markers: Luminal A (estrogen receptor alpha [ERα] and/or progesterone receptor [PR] positive, ErbB2 negative, Ki-67 low), luminal B (ERα and/or PR positive, ErbB2 positive or negative, and Ki-67 high), ErbB2-enriched (ERα and PR negative, ErbB2 positive, and Ki-67 high), and triple-negative (all markers negative and Ki-67 high) [24–27].

Conversely, the molecular subclassification of CMC remains ambiguous and is currently under investigation. Bergholtz *et al*. [28] demonstrated that gene expression profiles in CMTs closely resemble those of human breast tumors. Prior studies on CMC subtyping have largely drawn from HBC classification models, relying on the expression patterns of ERα, PR, and ErbB2 as determined by immunohistochemistry (IHC). IHC remains a widely adopted method due to its ability to distinguish protein expression in neoplastic versus adjacent normal tissue.

Although several CMC cell lines have been established, few have undergone comprehensive molecular classification. Most existing lines are derived from primary tumor tissues [26, 29–38]. Limitations in the specificity and sensitivity of antibodies for canine proteins further complicate accurate classification. While messenger RNA (mRNA) expression analysis has also been employed, inconsistencies between mRNA and protein levels have raised concerns regarding its reliability [32, 39]. To date, most characterized CMC cell lines fall within the triple-negative subtype, followed by ErbB2-enriched profiles (Table 1) [26, 29–38, 40,41]. Therefore, there is a critical need to develop a broader array of CMC cell lines that represent diverse molecular subtypes.

**Table 1:** Previously characterized CMC cell lines according to the protein expression levels of ERα, PR, and ErbB2.

Cell lines	Histology of origin	Samples	Molecular subtypes	References
CMT-7364	Intraductal papillary carcinoma	Primary tumor^a^, cells^b,c^	Triple-negative	[26]
IPC-366	Inflammatory mammary cancer	Primary tumor^a^, cells pellet^a^	Triple-negative	[29, 30]
FR37-CMT	Complex carcinoma	Primary tumor^a^	Triple-negative	[31]
UNESP-CM1	Solid carcinoma	Primary tumor^a^	ErbB2-enriched	[32]
		Cells^c^	n/a	
CMT-1	Complex carcinoma	Primary tumor^a^, cells^c,d^	ErbB2-enriched	[33]
REM134	Solid carcinoma	Cells^a^	ErbB2-enriched	[34–36]
CMT-U27	Simple carcinoma	Primary tumor^a^	n/a	[37]
SNU-CMG	Complex carcinoma	Primary tumor^a^	n/a	[38]
CMT-1026	Solid carcinoma	Primary tumor^a^	Triple-negative	[41]
		Cells^b^	n/a	

a IHC=Immunohistochemistry technique,

b ICC=Immunocytochemistry technique,

c IF=Immunofluorescence technique, ^d^WB=Western blot, CMC=Canine mammary cancer, ERα=Estrogen receptor alpha, PR=Progesterone receptor, ErbB2=Epidermal growth factor receptor 2 , CMT=Canine mammary tumor, n/a=Not available

Despite the growing interest in CMC as a comparative model for HBC, significant limitations persist in the availability and characterization of relevant *in vitro* systems. To date, most established CMC cell lines represent the triple-negative or ErbB2-enriched subtypes, which only partially reflect the heterogeneity observed in spontaneous CMTs. Moreover, comprehensive molecular subclassification of these cell lines remains incomplete, largely due to the scarcity of subtype-specific models and the limited availability of antibodies with high specificity and sensitivity for canine biomarkers. Although some studies have employed mRNA expression profiling for subtype determination, inconsistencies between mRNA and protein levels reduce the reliability of this approach. Consequently, few CMC cell lines have been classified using IHC, which remains the gold standard for subtype determination in both human and veterinary oncology. This underrepresentation of luminal A and luminal B subtypes poses a substantial barrier to the development of targeted therapies, limiting translational research potential and therapeutic exploration in hormone receptor-positive CMCs.

To address these limitations, the present study aimed to establish and characterize novel CMC cell lines representing the luminal A and luminal B molecular subtypes. These cell lines were derived from spontaneous malignant mammary tumors in dogs and subjected to comprehensive morphological, molecular, and functional analyses. The characterization included immunohistochemical profiling of key subtype markers (ERα, PR, ErbB2, and Ki-67), assessment of migratory behavior through wound healing and transwell assays, and evaluation of cancer-related gene expression (*EGFR, TP53, Bcl-2, PTEN, E-cadherin, N-cadherin*, and *SNAIL*) using reverse transcription quantitative real-time polymerase chain reaction (RT-qPCR). In addition, short tandem repeat (STR) profiling and Mycoplasma contamination testing were performed to authenticate the cell lines and ensure their suitability for future research. By providing robust, well-characterized models of luminal A and B CMC subtypes, this study seeks to expand the toolkit available for comparative oncology and facilitate the development of precision therapies for hormone receptor-positive CMTs.

## MATERIALS AND METHODS

### Ethical approval

This study received approval from the Committee on Animal Care and Use for Scientific Research at Kasetsart University (ACKU63-VET-027).

### Study period and location

This study was conducted from July 2020 to June 2021 at the Veterinary Hospital, Faculty of Veterinary Medicine, Kasetsart University.

### Tumor sample collection

CMT tissues were collected from 31 female dogs with mammary tumors larger than 2 cm in diameter who underwent surgical treatment at the Faculty of Veterinary Medicine Veterinary Hospital, Kasetsart University. Tumor tissues were sectioned, with a portion reserved for histopathological diagnosis. The remaining tumor was placed in 50 mL tubes containing 10 mL of DMEM-HG medium (DMEM High Glucose; Gibco, Cat# 11965-092, USA) supplemented with 1% antibiotic-antimycotic solution (Gibco, Cat# 15240-062) and chilled.

### Cell isolation and culture conditions

Thirty-one tumor tissues were washed with 20 mL of 1× Phosphate buffer saline (PBS) (Sigma-Aldrich, USA) supplemented with 1% antibiotic-antimycotic solution. The skin, connective tissue, and necrotic tissue were eliminated. Tissues were minced and washed with fresh medium until the suspension was clear. Subsequently, the tissue fragments were digested in 10–20 mL of 200 Units/mL Collagenase Type I (Gibco, Cat# 17100-017) in cell culture medium containing 25 mM HEPES (Gibco, Cat# 15630-080) and 1% antibiotic-antimycotic solution. Digestion was performed at 37°C in a shaking incubator (Vision Scientific, Model VS-8480SFN, South Korea) at 200 rpm for 2 h. After incubation, an equal volume of complete DMEM-HG medium (20% Fetal Bovine Serum (FBS) (Gibco, Cat# 10270-106) + 1% antibiotic-antimycotic solution) was added to stop the activity of collagenase type I enzyme, and the mixture was filtered through a 100 μm cell strainer (SPL Life Sciences, Cat# 93100, South Korea). The cell suspensions were centrifuged (Eppendorf, Model 5810R, Germany) at 400× *g* and 4°C for 10 min. Cell pellets were resuspended in 5 mL of complete DMEM-HG medium and seeded into a T25 Flask Filter Cap (SPL Life Sciences, Cat# SPL-70025, South Korea). Cultures were maintained in a CO^2^ incubator (Thermo Fisher Scientific, Model 4111, USA) at 37°C with 5% CO_2_.

Half of the culture medium volume was refreshed every other day throughout cultivation (up to 30 passages and more than 50 times of cumulative population doubling level [CPDL]). Fibroblasts were discarded by culturing cells in 250 μg/mL G418 (Geneticin Selective Antibiotic; Gibco, Cat# 10131035) in DMEM-HG containing 20% FBS until no fibroblast contamination remained. Then, 10 ng/mL epidermal growth factor (EGF) (Sigma-Aldrich, Cat# E4127) was added to the complete medium. The EGF was withdrawn after the 20^th^ passage. The FBS concentration was reduced to 10% beyond the 30^th^ passage.

### Cell maintenance and cryopreservation

For cell maintenance, approximately 200,000–300,000 cells were seeded in each T25 flask. Once the cell confluency reached 70%–80% of the total areas, the culture flask was gently rinsed twice with 3–5 mL of 1× PBS, treated with 1 mL of TrypLE Express enzyme (Gibco, Cat# 12604021, Denmark), and incubated at 37°C for 4–6 min. Subsequently, the enzyme was neutralized by adding an equal volume of the complete medium. The cell suspension was centrifuged at 400× *g* for 5 min, and the number of pellet cells was determined using a hemocytometer (Boeco, Hamburg, Germany). Cells were reseeded into cell culture flasks or cryopreserved as needed. For cryopreservation, cells were centrifuged, resuspended in cryopreservation medium (10% Dimethyl Sulfoxide (DMSO) (Sigma-Aldrich, Cat# D2650) in complete DMEM-HG with high serum, and transferred to cryogenic vials. Vials were stored at −80°C overnight and subsequently transferred to liquid nitrogen for long-term storage.

### Doubling time (DT) analysis

The initial (Ni) and harvested (Nh) cell numbers from each passage were used to calculate the cellular growth rate. DT values and CPDL were determined to assess the stability of the cell line. Primary cells cultured *in vitro* from the 30^th^ passage onward, which exhibited stable growth rates (DT values) and CPDL values exceeding 50, were considered successfully established cell lines [42]. At this stage, the cells exhibit consistent morphology, growth patterns, and viability.

### Cell line authentication

#### IHC

Both formalin-fixed paraffin-embedded (FFPE) samples of two novel CMC cell lines and their corresponding original tumor tissues were used for immunohistochemical subclassification. Both novel CMC cell lines (CMGT_071020 and CMGT_180321) were cultured in T75 flasks until they reached 80% confluence (approximately 5–10 million cells). Subsequently, these cells were trypsinized, centrifuged at 400× *g* for 10 min at 4°C, and the supernatant was removed. For FFPE processing, both pelleted cells and the corresponding tumor tissues were fixed in 10% buffered formalin at room temperature (25°C) overnight and subsequently embedded in paraffin. The FFPE samples were sectioned at 2.5 μm thickness, and tissue sections were mounted on positively charged microscope slides.

All FFPE sections were deparaffinized by incubating at 60°C for 60 min, followed by dewaxing, and rehydrating. Antigen retrieval was performed using epitope retrieval solution (Citrated pH 6 for ERα, PR, ErbB2, and Ki-67, and distilled water pH 7 for vimentin and multi-cytokeratin) in a steamer (Black and Decker, Model HS1000, Newark, DE, USA) for 40–60 min. Afterward, immunostaining was performed using the Novolink Polymer Detection System Kit (Leica Biosystems, Cat# RE7140-CE, Newcastle, UK) according to the manufacturer’s recommendation.

Primary antibodies against vimentin, multi-cytokeratin, ERα, PR, ErbB2, and Ki-67 were applied (Table 2). After primary antibody incubation, slides were treated with a post-primary and Novolink™-polymer, followed by 3 min exposure to 3,3’-Diaminobenzidine (DAB) chromogen (1:20 dilution) and counterstained with hematoxylin (C.V. Laboratories, Bangkok, Thailand). The slides were then air-dried and covered with a coverslip. As part of the experimental design, a normal canine uterus tissue was used as a positive control for ERα and PR. SKOV3 Human Ovarian Cancer cells (ATCC: HTB-77) were used as a positive control for ErbB2 expression (Supplementary Figure). For negative controls, the primary antibody was substituted with 1× tris-buffered saline.

**Table 2:** Primary antibodies for IHC.

Primary antibodies	Clone	Antibody dilution	Epitope buffer	Incubation IHC	Manufacturer
Vimentin	V9	Ready-to-use	DW pH 7	37°C for 60 min	Leica, UK
Muti-cytokeratin	AE1/AE3	Ready-to-use	DW pH 7	37°C for 60 min	Leica, UK
ERα	EP1	Ready-to-use	Citrate pH 6	4°C for O/N	Dako, Denmark
PR	Ab191138	1:70	Citrate pH 6	4°C for O/N	Abcam, US
ErbB2	A0485	1:100	Citrate pH 6	37°C for 60 min	Dako, Denmark
Ki-67	MIB-1	Ready-to-use	Citrate pH 6	37°C for 60 min	Dako, Denmark

IHC=Immunohistochemistry, ERα=Estrogen receptor alpha, PR=Progesterone receptor, ErbB2=Epidermal growth factor receptor 2 , DW=Distilled water, O/N = overnight

The stained slides were evaluated using a light microscope (Nikon, Model ECLIPSE Ci, China). Positive immunoreactivity was defined as brown DAB staining: cytoplasm for vimentin and multi-cytokeratin; nuclear for ERα, PR, and Ki-67, and membranous/cytoplasm for ErbB2. Protein expression levels were assessed using the Allred score. The Allred score ranged from 0 to 8 points [43]. Moderate-to-high staining (≥3.5) was considered positive. Within the positive range, the scores are further divided as follows: 3.5–5.5 is considered low positive (+1), 5.6–6.5 is considered moderate positive (+2), and 6.6–8.0 is considered high positive (+3).

For the interpretation of ErbB2 expression, guidelines set by the ASCO/CAP were adhered to assigning a score of 0 for none (no labeling), +1 for weak intensity (≤10% of cells), +2 for intermediate (>10% of cells), and +3 for strong intensity (>30% of cells). Only strong staining levels (+3) were considered indicative of positive [44–46].

The Ki-67 index, a marker of cellular proliferation, was assessed by counting the number of Ki-67-positive cells among the total number of neoplastic cells. This index provides information about the proportion of actively dividing cells within the tumor. The scores were evaluated in 10 randomly selected high-power fields (400×) using a Slide Scanner (Olympus, Model VS120, China). A cutoff value of 30% was used to differentiate high and low proliferation rates [47].

### *In vitro* functional assays

#### Wound healing assays

Wound healing assays were employed as a standard method to evaluate two-dimensional cellular migration *in vitro*. Two novel CMC cell lines (CMGT_071020 and CMGT_180321) and an ErbB2-enriched control cell line (REM134; ECACC: 12122002) were examined. CMGT_071020 (passages 50), CMGT_180321 (passages 43), and REM134 (passage 106) cells were seeded in 6-well plates in triplicate at a density of approximately 300,000–500,000 cells per well. The cells were cultured until reaching 100% confluency. Then, cell surfaces were lengthwise scraped using 3 mL Pasteur pipettes. After wounding, cells were incubated in a CO^2^ incubator at 37°C with 5% CO^2^ for 24 h. During this time, cellular migration into the wound area was monitored and photographed at serial time points of 0, 6, 12, 18, and 24 h using an inverted microscope (Nikon, Model ECLIPSE Ts2, China). Images were captured using NIS-Elements D imaging Software version 5.20 (Nikon, Tokyo, Japan) and analyzed to measure the reduction in intercellular gap over time.

The percentage of wound area was calculated using the following formula:

Wound area (%) = ([Space area at 6, 12, 18, and 24 h]/[Space area at 0 h]) × 100

#### Transwell migration assays

The transwell migration assay served as a technique used to study three-dimensional cell migration and assess the metastatic potential of cancer cells. In this assay, CMGT_071020 (passages 50), CMGT_180321 (passages 43), and REM134 (passage 106) cell lines were seeded at 5,000 cells per well into the upper chambers of transwell inserts (8 μm pore size) in triplicate. The upper chamber contained culture medium containing 5% FBS, while the lower chamber contained culture medium containing 10% FBS to serve as a chemoattractant. Cells were incubated for 24 h at 37°C with 5% CO_2_. After incubation, the culture media were removed from both chambers. The cells remaining on the upper surface of the membrane filter were gently scraped off with cotton moistened with fresh media. Subsequently, migrated cells on the underside of the membrane filter were fixed in ice-cold absolute methanol for 1 min, then stained with Modified Wright-Giemsa solution for 15 min, and rinsed with running tap water for 5 min. The number of migrated cells was quantified by counting five randomly selected fields under 40× magnification using an inverted microscope (Nikon, Model ECLIPSE Ts2, China).

### Molecular characterization

#### mRNA expression analysis

#### RNA purification, cDNA synthesis, and real-time RT-qPCR

Two novel CMC cell lines were harvested by trypsinization. Total RNA was extracted using a GeneJet RNA Purification Kit (Thermo Fisher Scientific, Waltham) according to the manufacturer’s protocol for adherent culture cells. The RNA concentration and purity were determined using a NanoDrop One/OneC Microvolume UV-Vis Spectrophotometer (Thermo Fisher Scientific, Waltham). To eliminate genomic DNA contamination, RNA samples were treated with RQ1 RNase-Free DNase (Promega, Madison, WI, USA). First-strand cDNA was synthesized using a SuperScript® III First-Strand Synthesis Kit (Invitrogen, Cat# 18080051, CA, USA). Briefly, 1 μg of total RNA was reverse transcribed in a 20 μl reaction containing 50 μM oligo(dT) primers, 10 mM dNTPs, and the cDNA synthesis mix. Reverse transcription was performed using a G-Storm GS482 PCR thermal cycler (Gene Technologies, Somerset, UK) under the following cycling conditions: 65°C for 5 min, 50°C for 50 min, 85°C for 5 min, and 37°C for 5 min. The relative mRNA expression levels of canine *EGFR, Bcl-2, TP53, PTEN, E-cadherin, N-cadherin*, and *SNAIL* were normalized to that of canine glyceraldehyde 3-phosphate dehydrogenase (*GAPDH*), which was selected as the reference gene due to its stable expression across various tissues and experimental conditions in canine cancer models [48–50]. The primer sequences are shown in Table 3. RT-qPCR was conducted in triplicate using a CFX96 Touch real-time PCR detection system (Bio-Rad, Hercules, CA, USA). The RT-qPCR protocol was performed as follows: initial denaturation at 95°C for 15 min, denaturation at 94°C for 15 s, annealing at 60°C for 30 s, and extension at 72°C for 30 s (40 cycles). Dissociation curves were generated between 65°C and 95°C to confirm the amplicon specificity. Gene expression levels were calculated using the 2^-ΔΔCq method [51].

**Table 3:** Primer sequences used for reverse transcription quantitative real-time polymerase chain reaction (RT-qPCR).

Gene	Primer sequences
*EGFR*	F: 5’- CGAGCACAAGGACAACATCG-3’
	R: 5’- CTCCACACATCGCTTTGGTG-3’
*TP53*	F: 5’- GCGGCCCAT CCTCACTATC-3’
	R: 5’- CACAAACGCGTACCTCAAAGC-3’
*Bcl-2*	F: 5’- TGGATGACTGAGTACCTGAA-3’
	R: 5’- GGCCTACTGACTTCACTT-3’
*PTEN*	F: 5’- AAAGCTGGAAAGGGACGAACTG-3’
	R: 5’- ACACATAGCGCCTCTGACTGGGT-3’
*SNAIL*	F: 5’- GACTCCCAGACTCGCAAGG-3’
	R: 5’- GACATGCGGGAGAAGGTTC-3’
*E-Cadherin*	F: 5’- TCCTGGGCAGGGTGAGTT-3’
	R: 5’- GAGGCCGCTTGACTGTAATC-3’
*N-Cadherin*	F: 5’- AGCACCCTCCTCAGTCAACG-3’
	R: 5’- TGTCAACATGGTCCCAG-3’
*GAPDH*	F: 5’- CCCACTCTTCCACCTTCGAC-3’
	R: 5’- AGCCAAATTCATTGTCATACCAGG-3’

*EGFR*=Epidermal growth factor receptor, *TP53*=tumor suppressor p53, *Bcl-2*=B-cell lymphoma 2, *PTEN* = Phosphatase and tensin homolog, *SNAIL*=*Snail* transcription factor, *GAPDH*=Glyceraldehyde 3-phosphate dehydrogenase

### Cell line verification

#### STR assay

STR profiling was performed to authenticate and genetically characterize cell lines by analyzing the unique patterns of repetitive DNA sequences that vary greatly between individuals. According to the manufacturer’s instructions, DNA samples were extracted from the pellet cells using Indispin® Pathogen kits (Indical Bioscience, Cat# SP54104, Germany). The isolated DNA was stored at −80°C and submitted to the Faculty of Veterinary Medicine, Kasetsart University, Kamphaeng Saen Campus for STR analysis. Sixteen microsatellite (STR) loci (FH2004, FH2097, FH2132, FH2608, FH2309, FH2161, FH2016, FH3619, FH2658, FH2010, FH2138, FH2145, FH2137, FH2973, FH2140, and FH2584) were used to confirm that each novel cell line was distinct.

#### Mycoplasma contamination detection

The mycoplasma DNA in the novel cell lines was investigated using a MycoSensor QPCR Assay Kit (Agilent Technology, Cat# 302106, USA) following the manufacturer’s recommendations. Determination was performed by quantitative real-time PCR (qPCR) using a CFX96 Touch Real-Time PCR Detection System (Bio-Rad, Hercules, USA).

### Statistical analysis

Data are presented as mean ± standard deviation (SD) from three independent replicate experiments. To assess statistical differences in variance, an unpaired t-test with Welch’s correction was used to analyze the relative mRNA expression level to compare the expression levels of each cancer-related gene between the two novel cell lines and to analyze the differences in the number of migrated cells among different cell types in independent experiments. A two-way analysis of variance with Tukey’s *post hoc* test for multiple comparisons was used to examine differences in the wound healing percentage at five different time points for the three cell lines. Statistical analysis was performed using GraphPad Prism version 10 (GraphPad Software, Inc, CA, USA), and *p*-value was considered indicative of a statistically significant difference. *p < 0.05; **p < 0.01; and ***p < 0.001.

## RESULTS

### Establishment of novel CMC cell lines

Thirty-one CMT tissues were obtained from the Faculty of Veterinary Medicine Veterinary Hospital, Kasetsart University (Table 4). Thirty-five percent of the samples (11/31) were diagnosed as benign. Sixty-five percent (20/31) of the cases were malignant tumors. Approximately 45% (9/20), 20% (4/20), 10% (2/20), 15% (3/20), and 10% (2/20) of these collected malignant tumors were simple carcinoma, complex carcinoma, sarcoma, carcinosarcoma, and solid carcinoma, respectively. The average age of dog patients with benign tumors was 10.6 ± 1.9 years, whereas dogs with malignant tumors had an average age of approximately 11.2 ± 2.2 years. Regarding breed, most CMT occurred in purebred dogs (over 50%), accounting for 55% of benign tumors (6/11) and 75% of malignant tumors (15/20). In addition, we found that more than 60% of CMT cases involved spayed dogs, comprising 64% benign (7/11) and 70% malignant (14/20) cases (Table 5).

**Table 4:** CMT collection.

No.	Code name	Breed	Age (years)	Neuter status	Histopathological diagnosis
1	CMGT_170720	Crossbreed	13	Neuter	Benign mixed tumor
2	CMGT_200720	Welsh terrier	13	Neuter	Papillary cystadenoma
3	CMGT_210720	Poodle	11	Intact	Benign mixed tumor
4	CMGT_290720	Shih Tzu	13	Neuter	Complex carcinoma
5	CMGT_070820	Crossbreed	8	Intact	Cystadenoma
6	CMGT_200820	Beagle	12	Neuter	Solid carcinoma
7	CMGT_090920	Bull Mastiff	8	Neuter	Papillary adenocarcinoma
8	CMGT_210920	Shih Tzu	13	Intact	Osteosarcoma
9	CMGT_011020	Crossbreed	10	Intact	Simple carcinoma
10	CMGT_071020	Siberian Husky	7	Neuter	Tubulopapillary carcinoma
11	CMGT_091120	Labrador	9	Neuter	Mammary adenoma
12	CMGT_101120	Crossbreed	10	Neuter	Hyperplastic
13	CMGT_031220-01	Pomeranian	13	Neuter	Malignant mixed tumor
14	CMGT_031220-02	Pomeranian	8	Intact	Malignant mixed tumor
15	CMGT_301220	Crossbreed	11	Neuter	Tubular adenoma
16	CMGT_260121	Golden retriever	13	Neuter	Ductal ectasia
17	CMGT_100221	Shih Tzu	11	Neuter	Adenocarcinoma
18	CMGT_110221	Shih Tzu	13	Intact	Mixed carcinoma
19	CMGT_220221	Shih Tzu	9	Neuter	Solid carcinoma
20	CMGT_240221	Yorkshire Terrier	8	Intact	Benign mixed tumor
21	CMGT_050321	Chihuahua	9	Neuter	Tubular ectasia
22	CMGT_100321	Crossbreed	12	Neuter	Complex carcinoma
23	CMGT_110321	Crossbreed	14	Intact	Simple carcinoma
24	CMGT_150321	Basset Hound	14	Neuter	Simple carcinoma
25	CMGT_170321	Maltese	12	Neuter	Carcinosarcoma
26	CMGT_180321	Yorkshire Terrier	9	Neuter	Papillary adenocarcinoma
27	CMGT_120521-01	Crossbreed	10	Intact	Complex carcinoma
28	CMGT_120521-02	Poodle	13	Neuter	Fibrosarcoma
29	CMGT_240521-01	Pomeranian	13	Neuter	Tubular carcinoma
30	CMGT_240521-02	Crossbreed	9	Neuter	Cystadenocarcinoma
31	CMGT_160621	Crossbreed	12	Intact	Papillary adenoma

CMT=Canine mammary tumors, CMGT=Canine mammary gland tumors

**Table 5:** The proportion of dog patients with CMTs according to age, breed, and spayed status.

Factors	Benign CMTs		Malignant CMTs	
	
	No. of cases	%	No. of cases	%
Age≤8	2	18.18	3	15.00
Age>8	9	81.82	17	85.00
Pure breed	6	54.55	15	75.00
Crossbreed	5	45.45	5	25.00
Intact	4	36.36	6	30.00
Spayed	7	63.64	14	70.00

CMT=Canine mammary tumors

**Figure 1:**
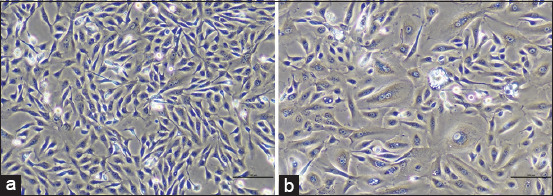
Morphology of 2 novel canine mammary cancer cell lines. (a) Tubulopapillary carcinoma: CMGT_071020 and (b) papillary adenocarcinoma: CMGT_180321. Amplification 10×. Scale bar = 100 μm. CMGT=Canine mammary gland tumors.

Two out of 31 (6.5%) samples were successfully developed into novel CMC cell lines, including CMGT_071020 and CMGT_180321, which originated from mammary tubulopapillary carcinoma (simple carcinoma) and papillary adenocarcinoma (simple carcinoma), respectively. All these novel cell lines underwent more than 30 passages, with calculated doubling times (DT) ranging from 1.2 to 2.2 days. To assess cell line stability, CPDLs were determined, resulting in CPDL values ranging from 85 to 278 times the cut point of 50 times (Table 6). These results indicate that our two primary cells developed into cell lines due to their stable growth rates (DT values), CPDL values exceeding 50 times, and the ability for continuous culture in the laboratory. During cultivation, we observed that CMGT_071020 cells exhibited a fibroblast-like (spindle-shaped) appearance, whereas CMGT_180321 cells displayed an epithelial-like morphology (Figure 1).

**Table 6:** Features of primary cancer cells that can grow *in vitro*.

Descriptions	Novel CMC cell lines	

	CMGT_071020	CMGT_180321
Age	7-year-old	9-year-old
Breed	Siberian Husky	Yorkshire Terrier
Sprayed status	Spayed	Spayed
Histopathological diagnosis	Tubulopapillary carcinoma	Papillary adenocarcinoma
Current Passage	105	52
DT values (days)	1.2	2.2
CPDL values (times)	287	85
Morphology	Fibroblast-like	Epithelial-like

CMC=Canine mammary cancer, CPDL=Cumulative population doubling level, DT=Doubling time, CMGT=Canine mammary gland tumors

### Molecular subclassification

In this study, the two novel CMC cell lines (CMGT_071020 and CMGT_180321) were used to examine the protein expression patterns using the IHC technique. Both novel CMC cell lines and their original tumor tissues were examined. The results indicated that IHC staining for PR and ErbB2 was negative in the original CMGT_071020 tumor. The other proteins, including vimentin, multi-cytokeratin, and ERα were positive in this sample. Meanwhile, the original tumor of CMGT_180321 was positive for all tested receptor protein markers, except ERα and ErbB2 were negative. The Ki-67 index for original tumors of CMGT_071020 and CMGT_180321 was 23% and 13%, respectively.

**Figure 2:**
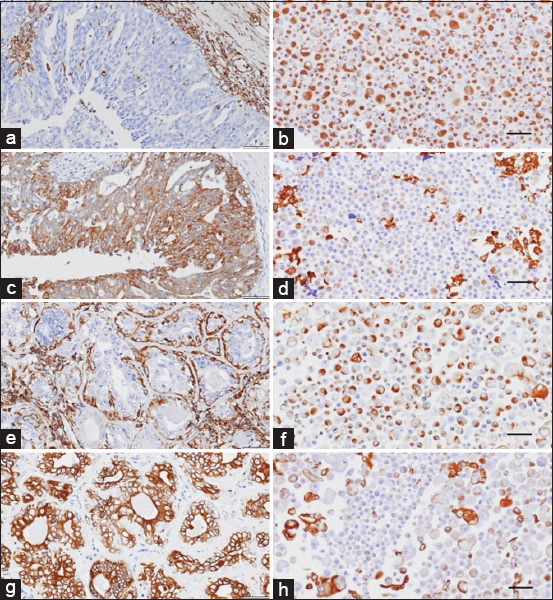
Immunohistochemistry (IHC) expression of vimentin and multi-cytokeratin in original tumor tissue and in both novel cell lines. IHC expression of original tumor tissue and CMGT_071020: (a and b) vimentin, (c and d) multi-cytokeratin. IHC expression of original tumor tissue and CMGT_180321, and (e and f) vimentin and (g and h) multi-cytokeratin in the original tumor (left) and novel cell lines (right). Amplification 40×. Scale bar=50 μm. CMGT=Canine mammary gland tumors.

**Figure 3:**
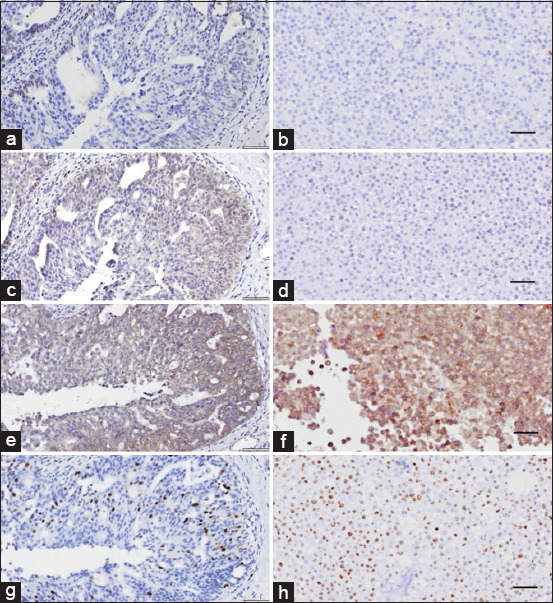
Immunohistochemistry (IHC) expression of canine mammary cancer markers in original tumor tissue and the CMGT_071020 cell line. IHC expression to (a and b) estrogen receptor alpha, (c and d) progesterone receptor, (e and f) epidermal growth factor receptor 2, and (g and h) Ki-67 in the original tumor (left) and CMGT_071020 cell line (right). Amplification 40×. Scale bar=50 μm. CMGT=Canine mammary gland tumors.

**Figure 4:**
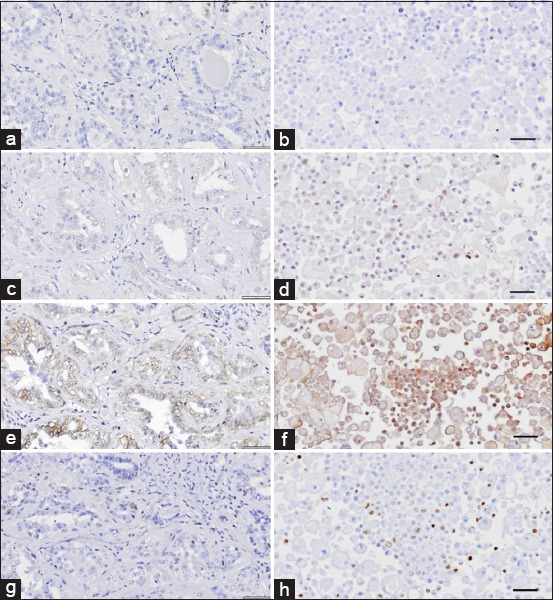
Immunohistochemistry (IHC) expression of canine mammary cancer markers in original tumor tissue and the CMGT_180321 cell line. IHC expression to (a and b) estrogen receptor alpha, (c and d) progesterone receptor, and (e and f) epidermal growth factor receptor 2, and (g and h) Ki-67 in the original tumor (left) and CMGT_180321 cell line (right). Amplification 40×. Scale bar = 50 μm. CMGT=Canine mammary gland tumors.

The IHC staining results of both novel CMC cell lines revealed that the CMGT_071020 cell line was positive for all tested markers, except for ERα. IHC staining for ERα and ErbB2 was negative in the CMGT_180321 cell line. The Ki-67 index for the CMGT_071020 and CMGT_180321 cell lines was 67% and 21%, respectively (Figures 2-4 and Table 7).

**Table 7:** Molecular subclassification of novel CMC cell lines.

Biomarker	CMGT_071020		CMGT_180321	
	
	Original tumor (n = 10)	Cell line (n = 10)	Original tumor (n = 10)	Cell line (n = 10)
Vimentin	Low	High	Low	High
Multi-cytokeratin	High	Moderate	High	High
ERα	Low	Negative	Negative	Negative
PR	Negative	Moderate	Low	Low
ErbB2	Negative	Positive	Negative	Negative
Ki-67 index	Low	High	Low	Low
	Luminal A	Luminal B	Luminal A	Luminal A

Slides were evaluated for protein expression levels using the Allred score and ASCO/CAP for ErbB2. Positivity within the score range of 3.5-8.0 was considered. Within this positive range, scores were further categorized as follows: 3.5-5.5 as low positive (+1), 5.6-6.5 as moderate positive (+2), and 6.6-8.0 as high positive (+3). Only high positive levels (+3) were interpreted as positive for ErbB2. CMC=Canine mammary cancer, ERα=Estrogen receptor alpha, PR=Progesterone receptor, ErbB2=Epidermal growth factor receptor 2 , CMGT=Canine mammary gland tumors

### *In vitro* functional assays

#### Wound healing assay

**Figure 5:**
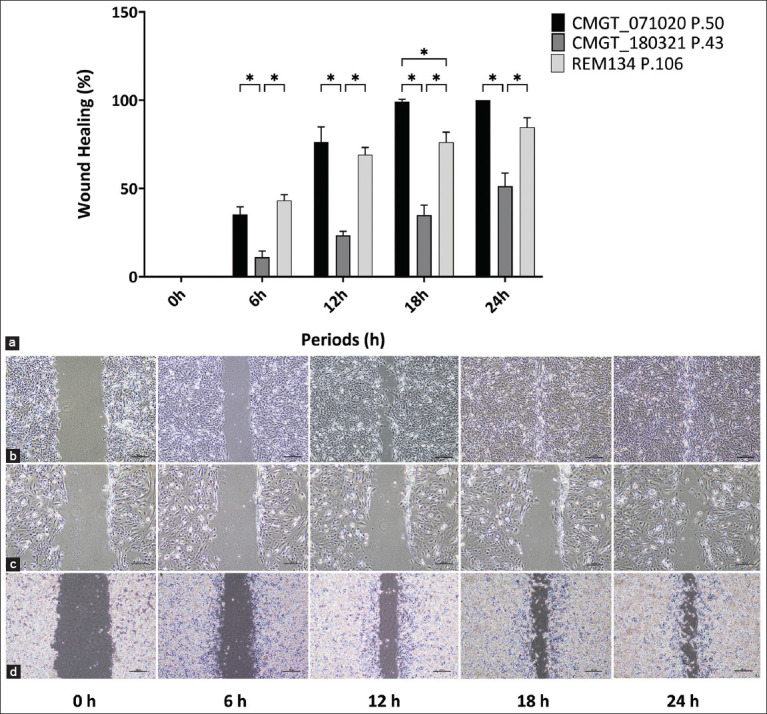
Wound healing ability and wound areas that change over time in the CMGT_071020, CMGT_180321, and REM134 cell lines. The *in vitro* movements and growths of two novel canine mammary cancer (CMC) cell lines (CMGT_071020 and CMGT_180321) were compared with commercial CMC cell lines (REM134) using the wound-healing assay. The percentage of the wound area was determined by measuring the change in the wound area at each time point relative to the initial wound area at 0 h. (a) Each data point represents the mean of triplicate experiments ± standard deviation. Statistical significance was assessed using a two-way analysis of variance followed by Tukey’s multiple comparisons *post hoc* tests in GraphPad Prism 10, with a p < 0.05 (*) considered indicative of a statistically significant difference, (b) area of wound healing in between 0 and 24 h of CMGT_071020, and (c) CMGT_180321, and (d) REM134 cell lines. Amplification 4×. Scale bar = 200 μm. CMGT=Canine mammary gland tumors.

Two-dimensional moving (2D) and growing between the two novel CMC cell lines (CMGT_071020 P.50 and CMGT_180321 P.43) and REM134 P.106 commercial CMC cell lines were compared (Figure 5). The CMGT_071020 and CMGT_180321 cell lines demonstrated 100% and 51% wound healing, respectively, in 24 h after scratching, At the same experimental period, REM134 exhibited a wound healing percentage of 85% of the total wound area.

#### Transwell migration assay

**Figure 6:**
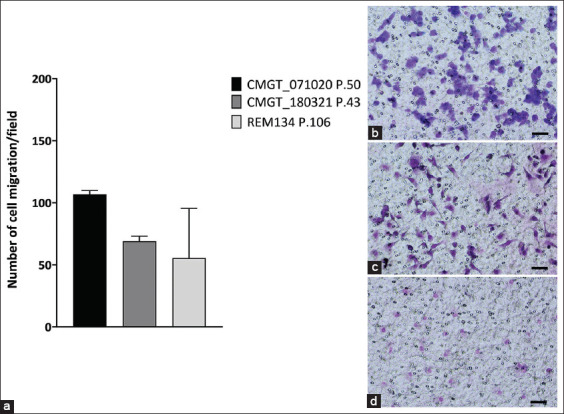
Cell migration ability using Transwell migration assay between CMGT_071020, CMGT_180321, and REM134 cell lines. Average number of migrated cells in 2 novel canine mammary cancer (CMC) and commercial CMC cell lines. (a) Each data point represents the mean of triplicate experiments ± standard deviation. Statistical significance was determined using an unpaired t-test with Welch’s correction in GraphPad Prism 10, with p < 0.05 (*), <0.01 (**), <0.001 (***) considered indicative of a statistically significant difference, (b) migrated cells of the CMGT_071020 P.50, (c) CMGT_180321 P.43, and (d) REM134 P.106 commercial CMC cell lines. The migrated cells were stained with Modified Wright-Giemsa stain. Amplification 10×. Scale bars = 100 μm. CMGT=Canine mammary gland tumors.

The average number of cells that migrated across the transwell membrane was calculated. We found that approximately 107, 69, and 56 migrated cells were identified on the Transwell filters of the CMGT_071020 (P.50), CMGT_180321 (P.43), and REM134 (P.106) cell lines, respectively. The results demonstrated that the CMGT_071020 cell line had significantly greater migratory ability than the CMGT_180321 cell line and showed a trend toward higher migratory ability than the REM134 cell line. However, due to the high SD observed in the REM134 measurements, these differences did not reach statistical significance when comparing the novel cell lines to the REM134 cell line (Figure 6).

### mRNA expression profiles of CMC genes

**Figure 7:**
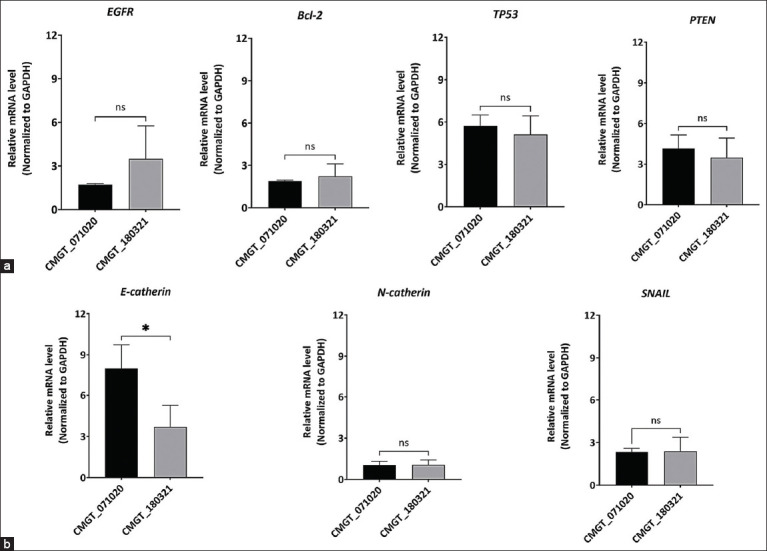
Gene expression profiles of two novel canine mammary cancer cell lines. (a) mRNA expression of four common cancer-related genes including *EGFR, Bcl-2, TP53*, and *PTEN*, and (b) EMT-related genes including E-cadherin, N-cadherin, and *SNAIL* in CMGT_071020 and CMGT_180321. Each data point represents the mean of triplicate experiments ± standard deviation. Statistical significance was determined using an unpaired t-test with Welch’s correction in GraphPad Prism 10, with p < 0.05 (*) considered indicative of a statistically significant difference. CMGT=Canine mammary gland tumors, *EGFR*=Epidermal growth factor receptor, *Bcl-2*=B-cell lymphoma 2, *TP53*=Tumor suppressor p53, *PTEN*=Phosphatase and tensin homolog.

The relative expression levels of four common cancer-related genes (*EGFR, TP53, Bcl-2*, and *PTEN*) and three EMT transcription factors (*E-cadherin, N-cadherin*, and *SNAIL*) were evaluated and compared between two novel cell lines using RT-qPCR. The relative mRNA expression levels of these genes in both CMT cell lines were normalized using canine *GAPDH*. The results indicated that *E-cadherin* expression was significantly higher in the CMGT_071020 compared to the CMGT_180321 cell line. In contrast, no significant differences were observed in the expression levels of the other six evaluated genes between the two novel CMT cell lines (Figure 7).

### Genetic authentication by DNA profiling

Using 16 microsatellite (STR) loci, the results indicated that the CMGT_071020 and CMGT_180321 cell lines are distinct from each other, with different DNA fingerprints, confirming their uniqueness (Table 8).

**Table 8:** DNA profile of novel CMC cell lines.

STR locus	Alleles called			

	CMGT_071020		CMGT_180321	
FH 2004	240	240	240	240
FH 2010	238	238	230	238
FH 2016	284	292	312	312
FH 2097	284	296	284	290
FH 2132	328	328	342	350
FH 2137	160	168	184	184
FH 2138	276	284	266	284
FH 2140	128	138	138	139
FH 2145	297	297	283	283
FH 2161	238	252	258	258
FH 2309	405	405	371	405
FH 2584	318	318	304	314
FH 2608	250	250	254	254
FH 2658	264	264	268	268
FH 2973	210	216	228	238
FH 3619	234	308	252	274

CMC=Canine mammary cancer, STR=Short tandem repeat, CMGT=Canine mammary gland tumors

### Mycoplasma screening of novel cell lines

All novel CMC cell lines were tested for mycoplasma contamination using the qPCR technique. We found that Ct values more than 40 cycles without Tm values were detected in all these cell lines. Mycoplasma testing was conducted on the successful establishment of the cell lines and was subsequently repeated as part of routine quality control, with testing conducted every 6 months during long-term culture.

## DISCUSSION

This study aimed to establish and characterize novel CMC cell lines representing luminal A and B subtypes.

### Need for diverse CMC cell lines and establishment efforts

CMC is the most prevalent cancer and a leading cause of death in female dogs. At present, effective treatments for CMC have not been successfully developed. Therefore, numerous studies have been conducted to identify more effective therapeutic strategies. One of the major challenges in CMC research is the limited diversity of available CMC cell lines. Although several CMC cell lines have been established and partially characterized (Table 1) [26, 29–38, 41], a critical need remains for a broader variety of well-defined models.

In this study, a total of 31 CMTs were collected from patient dogs. All tumors were pathologically diagnosed by board-certified Thai veterinary pathologists. Of these, approximately 65% were malignant and 35% were benign. The average age of dogs with benign tumors was 10.6 years, while that for malignant cases was 11.2 years. Purebred dogs were predominantly affected, accounting for 55% of benign and 75% of malignant tumors. These findings are consistent with a previous study by Burrai *et al*. [52], which reported a high prevalence of CMT in purebred dogs aged 9–12 years.

Over 60% of CMTs (both benign and malignant) in this study were detected in spayed dogs. This contrasts with a study by Mainenti *et al*. [53], in which approximately 60% of CMTs were found in non-spayed dogs and 40% in spayed ones. One explanation could be tumor recurrence post-surgery (with recurrence rates estimated at ~58%) [19]. Another possibility is that the spaying occurred after the first 2 years of life or beyond the initial four estrus cycles. Previous studies by Arendt and Kuperwasser [16], Burrai *et al*. [52], and Schneider *et al*. [54] have demonstrated that spaying within the first 2 years significantly reduces the risk of mammary tumors by over 70%.

The overall success rate for cell line development in this study was 6.5% (2/31). However, since 11 tumors were benign and generally poorly adaptable to *in vitro* culture, the effective success rate among malignant tumors was 10% (2/20). Two novel cell lines – CMGT_071020 and CMGT_180321 – were successfully established from malignant epithelial tumors (tubulopapillary carcinoma and papillary adenocarcinoma, respectively). The low success rate likely reflects challenges in the culture conditions. Some tumor cells require highly specific microenvironments or co-culture systems, especially for mixed mammary cancers. Furthermore, fibroblast overgrowth during early culture stages often hampers epithelial cell expansion, as fibroblasts rapidly proliferate and dominate [55, 56].

### Immunohistochemical subclassification of novel CMC cell lines

ERα, PR, ErbB2, and Ki-67 are critical biomarkers for CMC subclassification [57–59]. While mRNA expression levels are commonly assessed using RT-qPCR, and protein expression through Western blot and IHC, inconsistencies often arise due to post-transcriptional regulation. These regulatory processes may result in low protein levels despite high mRNA expression. Notably, few studies have subclassified CMC cell lines using IHC.

In this study, we classified the novel CMC cell lines using IHC and compared their biomarker expression patterns to those in their respective original tumors. Additional markers, including multi-cytokeratin and vimentin, were used to identify phenotypic characteristics. Remarkably, substantial variations were observed between the expression profiles in the cell lines and their original tumors. Specifically, an inverse relationship was noted in vimentin and multi-cytokeratin levels. While the original tumors showed high cytokeratin and low vimentin expression, the established cell lines exhibited higher vimentin and slightly reduced cytokeratin expression. This suggests that an epithelial-to-mesenchymal transition (EMT) occurred during *in vitro* adaptation, resulting in increased mesenchymal characteristics and decreased epithelial traits.

### EMT characteristics and migratory behavior

EMT is a key feature of cancer invasion, characte-rized by morphological changes from an epithelial to a mesenchymal phenotype and increased expression of mesenchymal markers (vimentin, N-cadherin, and fibronectin), along with decreased expression of epithelial markers (cytokeratin, E-cadherin, and β-catenin) [60–63]. To assess EMT and the aggressive potential of the novel cell lines, we evaluated mRNA expression of EMT-related genes (*E-cadherin*, *N-cadherin*, and *SNAIL*). Migration and invasion assays revealed that CMGT_071020 exhibited greater motility and invasiveness compared to CMGT_180321 and also displayed high *E-cadherin* expression.

Contrary to the conventional understanding that low E-cadherin marks EMT and malignancy, our findings suggest that CMGT_071020 may retain epithelial features while acquiring mesenchymal traits—a phenomenon known as partial EMT. This hybrid state has been linked to aggressive behavior in several cancer types. Further research on the functional role of E-cadherin and other EMT regulators is warranted to clarify their contributions in these cells [64].

### Expression of cancer-related genes

The relative expression levels of four common cancer-related genes (*EGFR, TP53, Bcl-2*, and *PTEN*) were analyzed in both luminal A and B cell lines. *EGFR* plays a role in pathways regulating proliferation, survival, and migration [14], while *TP53, Bcl-2*, and *PTEN* function as tumor suppressors, influencing cell cycle control, DNA repair, apoptosis, and oncogenic inhibition [11]. All four genes were expressed at comparable levels in both cell lines, indicating no significant alterations. Nevertheless, further gene profiling may uncover subtype-specific differences.

### Subtype classification and molecular divergence

Previous studies by Gama *et al*. [65], Varallo *et al*. [66], and Zheng *et al*. [67] report that most CMCs are of the luminal A (38%–40%) or luminal B (37%) subtype. In our study, CMGT_071020 was classified as luminal B (ERα-, PR+, ErbB2+, and Ki-67 high), whereas its original tumor was luminal A (ERα+, PR-, ErbB2-, and Ki-67 low). This discrepancy may reflect genetic alterations acquired during cell line development or variability in antibody specificity during IHC staining [68]. Conversely, CMGT_180321 and its original tumor were consistently classified as luminal A (ERα-, PR+, ErbB2-, and Ki-67 low) (Table 3).

Hormone receptor analysis showed low to nearly absent ERα expression, while PR expression ranged from low to moderate, consistent with prior findings that ERα is infrequently expressed in CMCs, whereas PR is detected in over 50% of cases [69, 70]. The CMGT_180321 line was ErbB2-negative, while CMGT_071020 was ErbB2-positive, aligning with Silva *et al*. [13], who reported ErbB2 positivity in 53% of CMCs.

The Ki-67 index was high in CMGT_071020 and its tumor, and low in CMGT_180321 and its tumor. Since high Ki-67 correlates with proliferation, this further supports the classification of CMGT_071020 as luminal B and CMGT_180321 as luminal A.

### Comparative migration analysis

Wound healing and transwell assays were used to compare the migration capacity of novel and commercial CMC lines. REM134, an ErbB2-enriched line [34], was used as a comparator. CMGT_071020 (luminal B) demonstrated significantly higher migration than CMGT_180321 (luminal A) and a trend toward greater migration than REM134. These findings suggest that luminal B subtypes may exhibit migratory and invasive behavior comparable to ErbB2-enriched cells, consistent with data from studies on HBC.

In HBC, luminal B tumors are more aggressive and associated with poorer prognosis than luminal A tumors. Yersal and Barutca [71] and Ades *et al*. [72] reported clinical similarities between luminal B-HBC and ErbB2-enriched HBC. ErbB2 expression appears to drive aggressive features in luminal B cancers. Further studies are necessary to explore the relationship between ErbB2 and its downstream pathways in CMC and confirm this association.

### Significance of the novel CMC cell lines

These novel luminal A and B CMC cell lines are valuable models for studying major hormone receptor-positive subtypes. While most existing CMC lines are triple-negative, the addition of these lines enables more accurate investigation of hormone-responsive cancers [15]. Given that hormone receptor-positive CMCs may respond to targeted therapy, understanding their molecular features, including cancer stem cell populations, may advance the development of precision treatments [73].

Future studies should further characterize CMGT_071020, CMGT_180321, and ErbB2-enriched cell lines through transcriptomic and proteomic profiling. Key pathways, such as PI3K and MDR1, which contribute to metastasis and treatment resistance, warrant particular attention. Although luminal B-HBC is known for worse outcomes compared to luminal A, this has yet to be fully demonstrated in CMCs.

To validate these findings, future work should include clinical investigations and *in vivo* xenograft models to evaluate tumorigenicity and metastatic behavior under physiological conditions. These models will provide essential preclinical data for the development of targeted therapies and the advancement of translational research in CMC.

## CONCLUSION

In this study, we successfully established and comprehensively characterized two novel CMC cell lines, CMGT_071020 and CMGT_180321, representing the luminal B and luminal A molecular subtypes, respectively. These cell lines were derived from malignant epithelial tumors and demonstrated stable, long-term growth beyond 30 passages, with doubling times ranging from 1.2 to 2.2 days and CPDL values exceeding 85. Immunohistochemical subclassification and mRNA expression profiling confirmed their distinct phenotypic identities, while functional assays revealed that the luminal B-cell line (CMGT_071020) exhibited significantly greater motility and invasiveness than its luminal A counterpart (CMGT_180321), suggesting a more aggressive biological behavior. Interestingly, partial EMT features were observed in both cell lines, likely induced by *in vitro* adaptation.

The strength of this study lies in the successful establishment of hormone receptor-positive CMC models, which are underrepresented among existing cell lines that predominantly reflect triple-negative or ErbB2-enriched phenotypes. These models enable subtype-specific investigations and provide critical platforms for comparative oncology, especially for evaluating CMC as a naturally occurring model of HBC.

From a practical standpoint, these novel cell lines offer valuable *in vitro* systems for studying tumor biology, metastatic mechanisms, and drug responsiveness in luminal-type CMCs. This is particularly relevant for developing targeted therapeutics tailored to hormone-responsive tumors, which may potentially improve treatment outcomes for both veterinary patients and comparative human oncology.

However, limitations include the relatively low establishment rate (6.5%) due to challenges such as fibroblast overgrowth, the requirement for specific growth conditions, and the inability of benign tumors to adapt to culture. In addition, subtle discrepancies between IHC profiles of the cell lines and their original tumors underscore the influence of culture-induced phenotypic drift.

Future research should focus on transcriptomic and proteomic profiling to elucidate the signaling networks underlying subtype-specific behaviors. The development of *in vivo* xenograft models will be critical for evaluating tumorigenic potential, metastatic capacity, and therapeutic responsiveness under physiological conditions. Investigating key pathways, such as PI3K/AKT and MDR1, and identifying cancer stem cell populations within these subtypes could further enhance their translational relevance.

CMGT_071020 and CMGT_180321 represent the first fully characterized luminal B and luminal A CMC cell lines, respectively. Their establishment addresses a significant gap in the field and provides essential tools for advancing comparative mammary oncology, precision medicine, and preclinical therapeutic development.

## DATA AVAILABILITY

The supplementary data can be made available from the corresponding author upon request.

## AUTHORS’ CONTRIBUTIONS

JJ: Conceptualization, methodology, data curation, investigation, visualization, writing–original draft, and writing–review and editing. WS: Conceptualization, funding acquisition, investigation, project administration, resources, supervision, validation, writing–original draft, and writing–review and editing. KS: Funding acquisition, investigation, project administration, resources, and supervision. UJ: Conceptualization, methodology, and resources. YP, KoS, SC, SP, RS, KW, CW, and NS: Methodology. NN and WiS: Sample collection. All authors have read and approved the final manuscript.
